# Influence of Selenium Pressure on Properties of AgInGaSe_2_ Thin Films and Their Application to Solar Cells

**DOI:** 10.3390/nano15151146

**Published:** 2025-07-24

**Authors:** Xianfeng Zhang, Engang Fu, Yong Lu, Yang Yang

**Affiliations:** 1 Guangzhou College of Technology and Business, Sanshui Compus, Foshan 528137, China; 2 State Key Laboratory of Nuclear Physics and Technology, School of Physics, Peking University, Beijing 100871, China; efu@pku.edu.cn; 3 School of Computer Science and Artificial Intelligence, Foshan University, Foshan 528231, China; luy_2021@fosu.edu.cn; 4 Aenerx-Technology Co., Ltd., Hangzhou 311121, China; gavin.yang@aenerx-tech.com

**Keywords:** AgInGaSe_2_ solar cell, selenium pressure, conversion efficiency

## Abstract

A wide-bandgap AgInGaSe_2_ (AIGS) thin film was fabricated using molecular beam epitaxy (MBE) via a three-stage method. The influence of Selenium (Se) pressure on the properties of AIGS films and solar cells was studied in detail. It was found that Se pressure played a very important role during the fabrication process, whereby Se pressure was varied from 0.8 × 10^−3^ Torr to 2.5 × 10^−3^ Torr in order to specify the effect of Se pressure. A two-stage mechanism during the production of AIGS solar cells was concluded according to the experimental results. With an increase in Se pressure, the grain size significantly increased due to the supply of the Ag–Se phase; the superficial roughness also increased. When Se pressure was increased to 2.1 × 10^−3^ Torr, the morphology of AIGS changed abruptly and clear grain boundaries were observed with a typical grain size of over 1.5 μm. AIGS films fabricated with a low Se pressure tended to show a higher bandgap due to the formation of anti-site defects such as In and Ga on Ag sites as a result of the insufficient Ag–Se phase. With an increase in Se pressure, the crystallinity of the AIGS film changed from the (220)-orientation to the (112)-orientation. When Se pressure was 2.1 × 10^−3^ Torr, the AIGS solar cell demonstrated its best performance of about 9.6% (Voc: 810.2 mV; Jsc: 16.7 mA/cm^2^; FF: 71.1%) with an area of 0.2 cm^2^.

## 1. Introduction

With the increasing requirement for renewable energy to reduce CO_2_ emissions, solar cells are being recognized as a potential substitution for traditional fossil fuels. In recent decades, the solar cell industry has developed very fast. Cu(In, Ga)Se_2_ (CIGS) solar cells, which have a chalcopyrite structure, are one of the most promising materials that can be used to fabricate high-efficiency thin film solar cells at a low cost [1,2,3]. It is very hard to continue increasing efficiency without increasing cost, since it takes 2 years for an increase of 0.29% conversion efficiency [4]. It has been reported that the solar cell’s conversion efficiency can be further increased via the fabrication of a tandem solar cell [5,6]; the bandgap requirement for top cells is around 1.7 eV [7]. Ag(In, Ga)Se_2_ (AIGS) thin films have been found to be a very suitable candidate for the top cell due to the following merits: (1) the bandgap of AIGS can be tuned by increasing the Ga/(Ga + In) atomic ratio from 1.24 eV (AgInSe_2_) to 1.83 eV (AgGaSe_2_), which satisfies the requirement for the bandgap of the top cell [8,9,10]; and (2) it has the same chalcopyrite structure as CIGS, leading to a low lattice mismatch between the bottom cell and the top cell. In our previous papers, AIGS thin films have been applied to the absorber layers of solar cells [11,12,13]. It has been suggested that the three-stage method is an effective approach for fabricating highly efficient CIGS solar cells [14,15,16]. To deposit AIGS films with a high conversion efficiency, a modified three-stage method using the molecular beam epitaxial system (MBE) has been developed, in which an efficiency of over 10% has been obtained [17].

The fabrication process for AIGS solar cells has been studied in detail. Extensive studies have been conducted to understand the influence of fabrication parameters on the properties of AIGS thin films. However, the effects of Se beam pressure on the electrical and optical properties of AIGS thin films are not yet well understood.

In this paper, AIGS thin films are fabricated in a molecular beam epitaxy (MBE) system. Four Knudsen cells (K-cells) filled with Ag, In, Ga, and Se are attached to the growth chamber. During the deposition process, the Se pressure varies from 0.8 × 10^−3^ Torr to 2.5 × 10^−3^ Torr, while the pressure of Ag, In, and Ga is maintained. The effect of the Se/(Ga + In) (Se/III) ratio on the electrical, optical, and structural properties of AIGS films has been studied. The as-fabricated AIGS thin films are further deposited onto other layers to complete the solar cells; solar cell performances with various Se/III ratios in AIGS films have been investigated.

## 2. Experimental Methods

### 2.1. Fabrication of AIGS Films

A three-stage method, using MBE apparatus under ultra-high vacuum conditions (<10^−5^ Pa), was adopted to deposit AIGS films on Mo-coated soda-lime-glass (SLG) substrate, which has been introduced in our other papers [18]. The MBE chamber was equipped with 4 K-cells filled with Ag, In, Ga, and Se, respectively. During the deposition process, materials were evaporated from the cells, and the pressure was controlled separately. All source materials were of 5N purity. During the first stage, In, Ga, and Se were deposited on the substrate at a temperature of 400 °C for 40 min. In the second stage, the substrate temperature increased to 580 °C in order to deposit Ag and Se, which lasted for 40 min. Finally, In, Ga, and Se were deposited again for 5 min to obtain AIGS thin films with a stoichiometric composition. The final thickness of the AIGS absorber layer was about 2 μm.

The structure of the AIGS solar cell comprises Mo/AIGS/CdS/ZnO(B)/Al. Other layers of the solar cell were fabricated to complete a whole solar cell structure. The CdS buffer layer was deposited using a chemical bath deposition method with a thickness of 50 nm. The ZnO and B-doped ZnO layers were deposited on the CdS layer via a metal–organic chemical vapor deposition with a thickness of 80 and 600 nm, respectively. Finally, the front contact of the Al grid was deposited on top using an evaporation method.

### 2.2. Characterization Methods

The as-deposited AIGS thin films were removed from the MBE growth chamber and cut into small pieces to carry out a scanning electron microscope (SEM; JSM-7001F, JEOL Ltd., Tokyo, Japan) measurement with an accelerating voltage of 5 KeV. The surface morphology was analyzed by SEM. An energy-dispersive X-ray spectrometer (EDS; JED-2300T, JEOL Ltd., Japan) attached to the SEM equipment was used to analyze the composition of the AIGS thin film. An X-ray diffractometer (XRD; Rigaku Hyper-RINT, Tokyo, Japan) with Cuα radiation was used to characterize the crystallinity of the AIGS films, with an operating voltage and current of 40 kV and 20 mA, respectively. The solar cell performance was measured using a 913 CV type I-V tester, which was illuminated by an EKO (LP-50B) solar simulator with an intensity of 100 mW/cm^2^ (AM1.5). In order to obtain the standard illumination density, the simulator was calibrated with a standard Si solar cell. The quantum efficiency (QE) of the AIGS solar cell was characterized using a QE tester (QE-2000, Otsuka Electronics Co., Ltd., Osaka, Japan). A GaAs standard was used to calibrate the system.

## 3. Results and Discussion

### 3.1. Composition and Surface Morphology of AIGS Films

**Figure 1:**
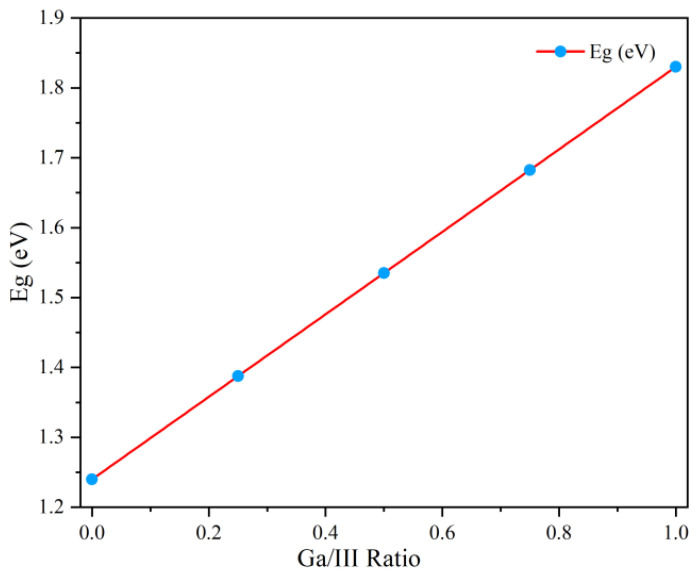
The relationship between the Ga/III ratio and the bandgap of AIGS films.

Table 1 shows the influence of Se pressure on the composition of the AIGS films. It can be seen that the Se/III ratio markedly increases with the increase in Se pressure. Additionally, the Ag/III ratio in the AIGS films tends to be low when a low Se pressure is used. With the increase in Se pressure, the Ag/III ratio gradually increases; the reason for this can be attributed to the formation of the Ag-deficient phase. A similar phenomenon has been reported in CIGS [19]. In addition, the Ga/III ratio varies to a small extent. Figure 1 shows the relationship between the Ga/III ratio and the bandgap of AIGS films. It can be concluded that the above AIGS films show a bandgap between 1.73 eV and 1.75 eV; when the Se pressure is 2.1 × 10^−3^ Torr, the bandgap of the AIGS films is close to the theoretical value of 1.72 eV. These samples were further researched and used to fabricate AIGS solar cells; the results will be discussed in the following sections.

**Figure 2:**
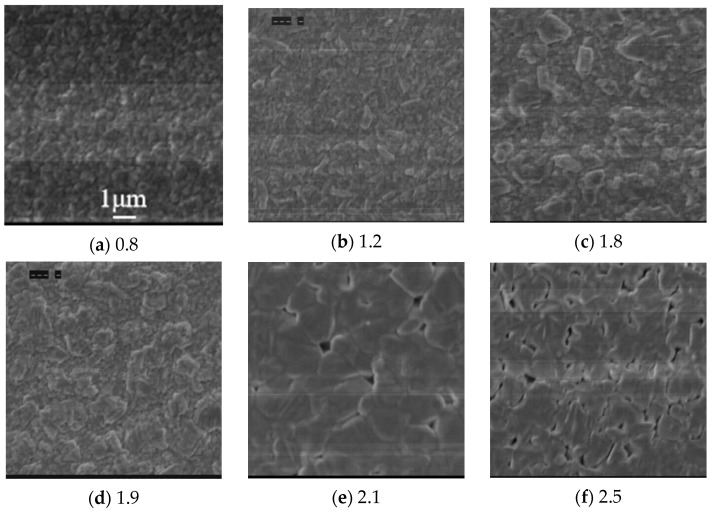
Dependence of surface morphology on Se pressure (**a**) 0.8 × 10^−3^ Torr; (**b**) 1.2 × 10^−3^ Torr; (**c**) 1.8 × 10^−3^ Torr; (**d**) 1.9 × 10^−3^ Torr; (**e**) 2.1 × 10^−3^ Torr; (**f**) 2.5 × 10^−3^ Torr.

Figure 2a–f shows the dependence of the surface morphology of AIGS films on Se pressure. All the figures used the same scale bar as shown in Figure 2a. When Se pressure was 0.8 × 10^−3^ Torr, the AIGS film showed a grain size of 0.3 μm. As the Se pressure increased to 1.2 × 10^−3^ Torr, the grain size began to increase, and the grain changed from a circular shape to a strip shape. With the increase in Se pressure to 1.8 × 10^−3^ Torr, the grain size continued to increase, and the superficial roughness increased significantly. As the Se pressure increased to 1.9 × 10^−3^ Torr, the grains increased continuously, with large grains connecting to form island-like grains. The grain boundary was difficult to detect. However, when the Se pressure was increased to 2.1 × 10^−3^ Torr, the morphology of the AIGS film changed abruptly and clear grain boundaries were observed. The typical grain size was over 1.5 μm. The film showed a dense and flat surface due to its stoichiometric composition, which was a typical AIGS surface morphology according to our former research [18]. When the Se pressure further increased to 2.5 × 10^−3^ Torr, the quality of the surface morphology deteriorated significantly. The grain size decreased, and the film showed more pinholes, which was supposedly caused by the evaporation of Se.

This process can be explained using the following reactions:

In + Ga + Se→(In, Ga)_2_Se_3_ (1)

During the first stage, In, Ga, and Se reacted to form a (In, Ga)_2_Se_3_ compound layer.

Ag + Se→Ag_2_Se (2)

(In, Ga)_2_Se_3_ + Ag_2_Se→Ag(In, Ga)Se_2_ (3)

Ag(In, Ga)Se_2_ (Ag rich) + (In, Ga)_2_Se_3_→Ag(In, Ga)Se_2_ (Ag poor) (4)

In the second stage, Ag and Se formed a Ag–Se compound. Then, (In, Ga)_2_Se_3_ and Ag_2_Se continued to react to form the Ag(In, Ga)Se_2_ layer. During the third stage, Ag(In, Ga)Se_2_ reacted with (In, Ga)_2_Se_3_ again to obtain the final Ag-poor AIGS films. It was observed that Se pressure played a very important role during the second stage, due to the formation of the crucial intermediate phase of Ag_2_Se. Thus, the quality of the AIGS films was affected significantly by Se pressure.

### 3.2. Optical Properties of AIGS Films with Different Se/III Ratios

Optical absorption measurements were carried out to calculate the bandgap of the AIGS solar cells. The real bandgap can be obtained from the following expression, combined with the photon absorption of the AIGS films:

(αhν)^2^ = *K*(hν − Eg) (5)

where α, h, ν, and Eg represent the absorption co-efficient, Planck’s constant, the light frequency, and the bandgap, respectively [20,21].

**Figure 3:**
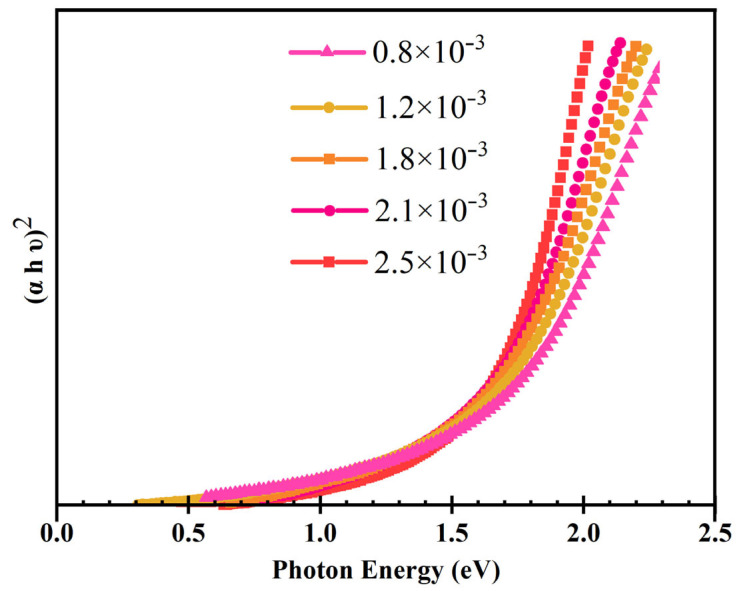
The (αhν)^2^ curve of the AIGS films as a function of incident photon energy with different Se pressures.

Figure 3 shows (αhν)^2^ as a function of photon energy. The intersection between the x-axis and the extension of the linear region of the absorption edge illustrates the bandgap of AIGS films. It can be concluded that AIGS films with a low Se/III atomic ratio (low Se pressure) tend to report a higher bandgap. With the increase in the Se/III atomic ratio, the bandgap of the AIGS films decreased markedly. Table 2 shows the approximate bandgap of AIGS films fabricated with different Se pressures. 

Since Ag and Se form a Ag–Se compound in the second stage, the lack of Se leads to a deficiency of Ag in the AIGS films. Thus, one of the possible reasons for this result is the formation of anti-site defects such as In and Ga on the Ag sites due to the impediment of the reaction (2). In this research, it was characterized that the Ag/III atomic ratio increased with the increase in Se pressure, although the Ga/III atomic ratio was maintained for different AIGS films. Thus, In_Ag_ or Ga_Ag_ defects are likely to exist in AIGS films with lower Se/III atomic ratios, which lead to an increase in the AIGS bandgap.

### 3.3. Influence of Se Pressure on the Structure of AIGS Films

**Figure 4:**
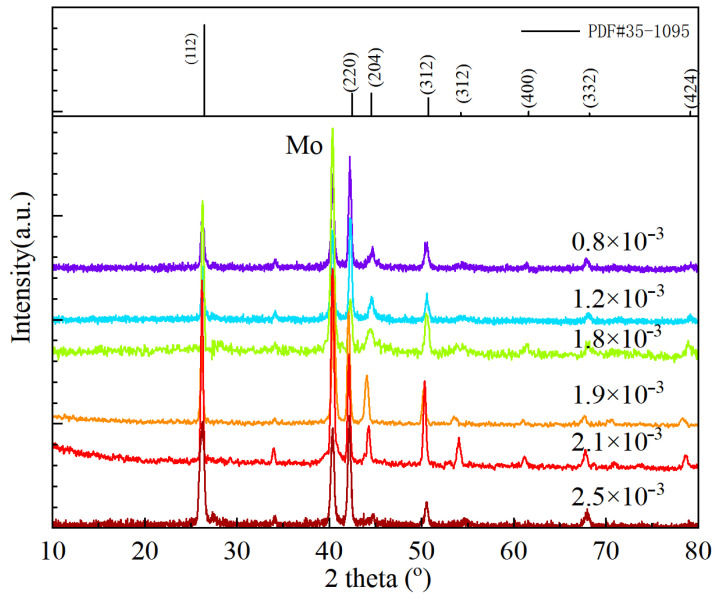
XRD patterns of AIGS solar cells with different Se pressures.

The XRD patterns of the above films are shown in Figure 4. The XRD curves show patterns of typical chalcopyrite AIGS films. Judging from the figure, all the XRD patterns exhibit four main peaks at around 2θ = 26.3°, 42.4°, 44.6°, and 56.5°, corresponding to the peaks of (112), (220), (204), and (312) of the AIGS film, respectively [22,23]. Moreover, the peak at 2θ = 40.4° is related to the crystallization of the Mo back contact layer. In addition, a slight shift in the AIGS peaks to higher 2θ angles was observed at higher Se pressures. This peak shift is probably caused by the increase in the Ag-deficient phase.

**Figure 5:**
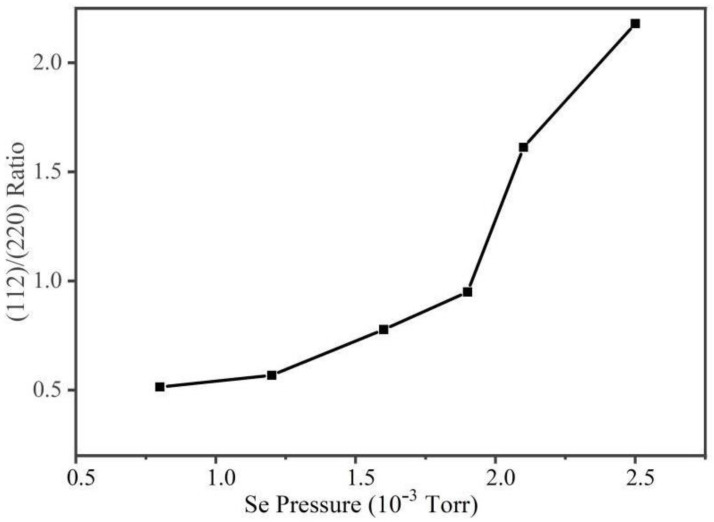
XRD peak intensity ratio of AIGS (112) and (220).

To analyze the preferred orientation of AIGS crystallization, the (112)/(220) peak intensity ratio as a function of Se pressure is shown in Figure 5. With the increase in Se pressure, a substantial increase in the (112)/(220) peak ratio is observed. When the Se pressure is 0.8 × 10^−3^ Torr, the main peak is (220). With the increase in Se pressure, the (220) peak decreases and the (112) peak increases, indicating the variation in the preferred orientation of AIGS films. When the Se pressure is over 1.9 × 10^−3^ Torr, the dominant peak changes to (112), which is typical for normal AIGS films that show a high performance. This result can be explained by the reaction during the deposition process. As explained previously, when Se pressure is low, the growth of the AIGS film is insufficient and the growth of the (112) peak is suppressed. With the increase in Se pressure, the growth of both the AIGS film and (112) is promoted, which concurs with our previous study [11]. When Se pressure is low, the formation of the Ag–Se phase is insufficient, and the Ag-rich phase is not enough. Thus, the AIGS films show a strong (220) peak due to the insufficient supply of Ag. On the other hand, Ag supply is enhanced with the increase in Se pressure and the growth of peak (112) is promoted as well. It can also be judged that the peak orientation is also related to the surface texture of AIGS films. When the grain size is small, the AIGS film has a (220)-preferred orientation. The dominant peak changes to (112) with the increase in grain size.

### 3.4. Influence of Se Pressure on the Performances of AIGS Films

**Figure 6:**
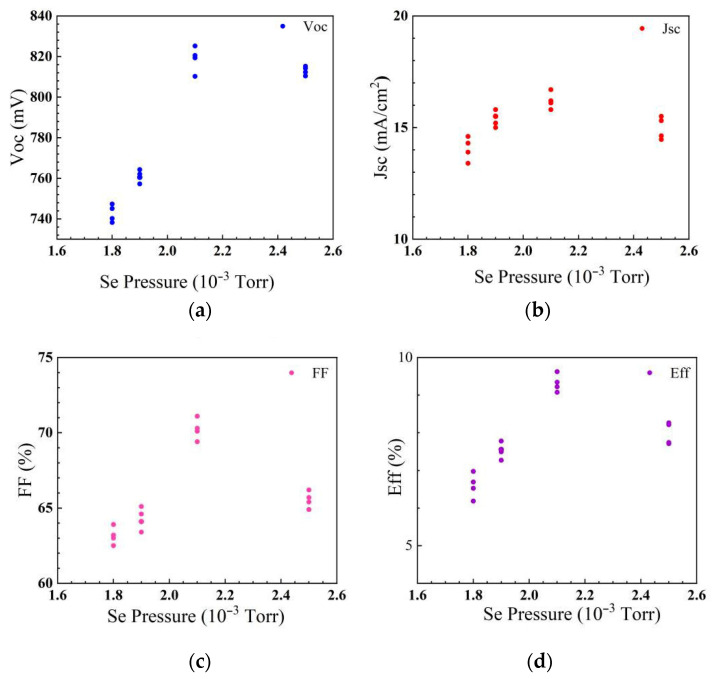
Performance parameters. (**a**) Open-circuit voltage (Voc); (**b**) short-circuit current density (Jsc); (**c**) fill factor (FF); (**d**) conversion efficiency of AIGS solar cells as a function of Se pressure.

The obtained AIGS films were then fabricated with other layers in order to obtain a full solar cell structure. To make the research more meaningful, AIGS solar cells fabricated with a Se pressure of over 1.2 × 10^−3^ Torr were used for further study, whereby the solar cell performance was characterized under standard conditions with an irradiation density of 100 mW/cm^2^. During the measurement, the intensity of the solar illumination was calibrated using a high-precision mono-crystalline Si solar cell to achieve a standard value. Figure 6 shows the photovoltaic characteristics of AIGS solar cells fabricated under different Se pressures with an area of 0.2 cm^2^. To reduce system errors and the influence of surrounding circumstances, the same measurement was conducted on four samples with the same deposition process. When the Se pressure was 1.8 × 10^−3^ Torr, the conversion efficiency of the AIGS solar cell was about 6.5%, with an open-circuit voltage (Voc) of about 745 mV, a short circuit current (Jsc) of 13.9 mA/cm^2^, and a fill factor (FF) of 63.1%. With the increase in Se pressure, the performance of the AIGS solar cell markedly increased. When the Se pressure was 2.1 × 10^−3^ Torr, the solar cell showed its best conversion efficiency of about 9.6% (Voc: 810.2 mV; Jsc: 16.7 mA/cm^2^; FF: 71.1%). However, when the Se pressure was increased to 2.5 × 10^−3^ Torr, the performance of the AIGS solar cell decreased to around 8.2% (Voc: 810.4 mV; Jsc: 14.6 mA/cm^2^; FF: 66.2%). It was inferred that the poor performance of the solar cell with a low Se pressure was related to the low quality of the AIGS film as a result of the insufficient growth of AIGS crystallinity. With the increase in Se pressure, the growth of the AIGS film was promoted and the crystallinity of the absorber layer was improved, which led to better solar cell performance. The AIGS solar cell showed the highest conversion efficiency of around 9.6% when the Se pressure was 2.1 × 10^−3^ Torr, indicating the best deposition parameter. However, when the Se pressure further increased, the solar cell performance began to deteriorate because of the low superficial quality caused by Se evaporation from AIGS films.

**Figure 7:**
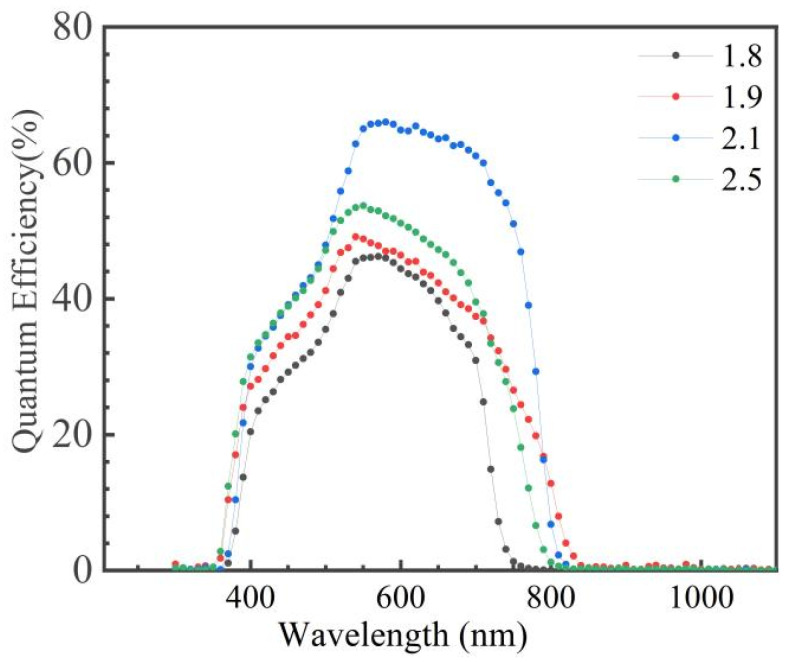
QE of AIGS solar cells with different Se pressures (×10^−3^ Torr).

Figure 7 shows the external quantum efficiency (QE) curves of the AIGS solar cells with different Se pressures. The QE of the solar cell fabricated with a high Se pressure was higher than that of the ones fabricated with low Se pressures. The absorption edges at the short wavelength side of 510 nm and 380 nm corresponded to the CdS and ZnO layers [23,24,25], respectively, which were common when using the CdS buffer and ZnO window layers. The variation in absorption referred to the difference in quality of the CdS and ZnO films. The QE curve shows an abrupt drop at the long wavelength side around 770 nm, which was attributed to the AIGS absorption edge. These results are in accordance with the solar cell’s performance.

**Figure 8:**
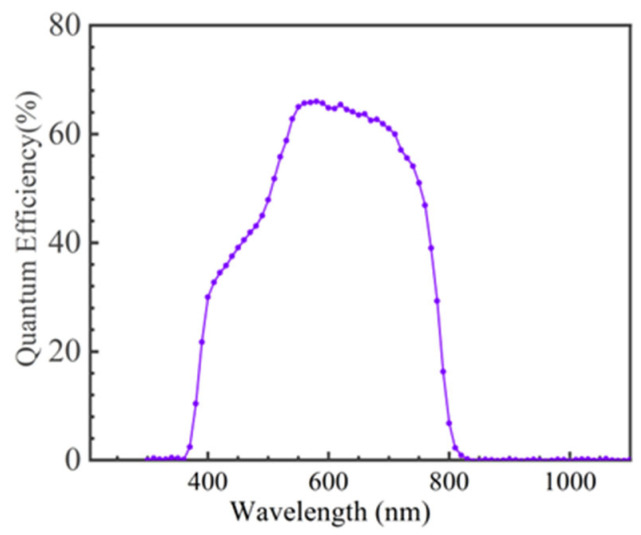
QE curve of the AIGS solar cell with the best conversion efficiency.

Figure 8 shows the QE curve of the AIGS solar cell with the best conversion efficiency.

Based on the EQE data of the solar cell, the Jsc can be calculated using the following expression [26]:

(6) $$J_{sc}=q\int_{0}^{\infty }QE\left(E\right)b_{s}\left(E,T_{s}\right)dE,$$

where *q* is the unit charge of the electron, *QE* is the quantum efficiency, and *b_s_* is the solar irradiation. The sunlight spectrum data can be obtained from [27] for an air mass of 1.5 on Earth. Based on Equation (6), Figure 8, and the solar irradiation spectrum, the J_SC_ of the AIGS solar cell was calculated as 16.5 mA/cm^2^ with a Se pressure of 2.1 × 10^−3^ Torr. It can be seen that the J_SC_ calculated from the *QE* curve is almost the same as the value measured using the I-V curve.

## 4. Conclusions

A MBE three-stage method was used to fabricate AIGS thin films, which were further used to obtain a complete solar cell structure. The influence of Se content on the AIGS thin films and the solar cells’ performances was evaluated by changing the Se pressure from 0.8 × 10^−3^ Torr to 2.5 × 10^−3^ Torr during the fabrication of AIGS precursors. The grain size, superficial morphology, and crystallization of the AIGS films were significantly influenced by Se pressure. With an increase in Se pressure, the grain size increased significantly and the best film was obtained when the Se pressure was increased to 2.1 × 10^−3^ Torr with a typical grain size of over 1.5 μm. AIGS films fabricated with a low Se pressure tended to show a higher bandgap due to the formation of anti-site defects such as In and Ga on the Ag sites due to an insufficient Ag–Se phase. With an increase in Se pressure, the AIGS films changed from a (220)-orientation to a (112)-orientation crystallinity. The highest solar cell conversion efficiency of 9.6% (Voc: 810.2 mV; Jsc: 16.7 mA/cm^2^; FF: 71.1%) with an area of 0.2 cm^2^ was obtained when the Se pressure was 2.1 × 10^−3^ Torr. The bandgap of the AIGS thin films was calculated as 1.65 eV, according to the QE curve of the AIGS solar cell.

## Figures and Tables

**Table 1:** Dependence of composition on Se pressure.

Se Pressure (10^−3^ Torr)	Ag	In	Ga	Se	Ag/III	Ga/III	Se/III
0.8	17.08	4.90	27.75	50.27	0.52	0.85	1.54
1.2	19.10	4.02	26.09	50.79	0.63	0.87	1.69
1.8	19.19	4.36	25.33	51.12	0.61	0.85	1.72
1.9	19.35	4.04	23.83	52.78	0.69	0.86	1.89
2.1	20.05	4.37	21.90	53.68	0.76	0.83	2.04
2.5	19.16	4.31	21.86	54.67	0.73	0.84	2.09

**Table 2:** Bandgap of AIGS films with different Se pressures.

Se Pressure (10^−3^ Torr)	0.8	1.2	1.8	2.1	2.5
Eg (eV)	1.70	1.66	1.61	1.58	1.57

## Data Availability

The raw data supporting the conclusions of this article will be made available by the authors on request.
